# Uterine Notch2 facilitates pregnancy recognition and corpus luteum maintenance via upregulating decidual Prl8a2

**DOI:** 10.1371/journal.pgen.1009786

**Published:** 2021-08-30

**Authors:** Haili Bao, Yang Sun, Ningjie Yang, Na Deng, Zhangli Ni, Yedong Tang, Gaizhen Li, Lili Du, Yan-Ling Wang, Dunjin Chen, Haibin Wang, Shuangbo Kong

**Affiliations:** 1 State Key Laboratory of Stem Cell and Reproductive Biology, Institute of Zoology, Chinese Academy of Sciences, Beijing, China; 2 University of Chinese Academy of Sciences, Beijing, China; 3 Fujian Provincial Key Laboratory of Reproductive Health Research, School of Medicine, Xiamen University, Xiamen, China; 4 Department of Obstetrics and Gynecology, The Third Affiliated Hospital of Guangzhou Medical University, Guangzhou, China; 5 Department of Obstetrics and Gynecology, The First Affiliated Hospital of Xiamen University, Xiamen, China; Université de Montréal - Faculté de Médecine Vétérinaire, CANADA

## Abstract

The maternal recognition of pregnancy is a necessary prerequisite for gestation maintenance through prolonging the corpus luteum lifespan and ensuring progesterone production. In addition to pituitary prolactin and placental lactogens, decidual derived prolactin family members have been presumed to possess luteotropic effect. However, there was a lack of convincing evidence to support this hypothesis. Here, we unveiled an essential role of uterine Notch2 in pregnancy recognition and corpus luteum maintenance. Uterine-specific deletion of *Notch2* did not affect female fertility. Nevertheless, the expression of decidual Prl8a2, a member of the prolactin family, was downregulated due to *Notch2* ablation. Subsequently, we interrupted pituitary prolactin function to determine the luteotropic role of the decidua by employing the lipopolysaccharide-induced prolactin resistance model, or blocking the prolactin signaling by prolactin receptor-Fc fusion protein, or repressing pituitary prolactin release by dopamine receptor agonist bromocriptine, and found that *Notch2*-deficient females were more sensitive to these stresses and ended up in pregnancy loss resulting from abnormal corpus luteum function and insufficient serum progesterone level. Overexpression of Prl8a2 in *Notch2* knockout mice rescued lipopolysaccharide-induced abortion, highlighting its luteotropic function. Further investigation adopting *Rbpj* knockout and DNMAML overexpression mouse models along with chromatin immunoprecipitation assay and luciferase analysis confirmed that Prl8a2 was regulated by the canonical Notch signaling. Collectively, our findings demonstrated that decidual prolactin members, under the control of uterine Notch signaling, assisted pituitary prolactin to sustain corpus luteum function and serum progesterone level during post-implantation phase, which was conducive to pregnancy recognition and maintenance.

## Author summary

Progesterone secreted from the corpus luteum in the ovary is indispensable to pregnancy maintenance in both rodents and humans. Therefore, prolonged corpus luteum lifespan and sustainable progesterone production is a prerequisite for a successful pregnancy. In rodents, in addition to pituitary prolactin and placental lactogens, decidual derived factors have been presumed to possess luteotropic effects during the post-implantation stage. In this study, utilizing a mouse model with uterine specific deletion of Notch2, which displayed decreased level of decidual prolactin member Prl8a2, combined with multiple approaches to interrupt the pituitary prolactin signal, we demonstrated that decidual derived Prl8a2 assisted pituitary prolactin to sustain corpus luteum function and serum progesterone level during post-implantation phase, which was conducive to pregnancy recognition and maintenance. In addition, the expression of decidual Prl8a2 was under the direct control of the canonical Notch pathway. Together, we herein provide convincing evidence that decidual produced Prl8a2, modulated by uterine canonical Notch signaling, exhibits luteotropic functions and contributes to pregnancy maintenance.

## Introduction

The successful establishment and maintenance of pregnancy relies on the elaborately regulated fetal-maternal crosstalk in the uterus as well as the coordinated adaptation of other maternal organs. Sustainable corpus luteum (CL) function and continuous progesterone (P4) secretion, which is pivotal to gestation maintenance, necessitates the maternal recognition of pregnancy as a prerequisite [1–3].

Mechanisms underlying pregnancy recognition vary considerably among species. In rodents, granulosa cells transform into morphologically and functionally distinct CL after ovulation. In the absence of mating, the CL regresses rapidly so that the next estrus cycle proceeds. If mating occurs, the cervical stimulation evokes the release of prolactin (Prl) from the anterior pituitary, which is able to support the CL for 10–12 days [4]. The further extension of CL lifespan throughout gestation requires the presence of conceptus [5]. With embryonic development and placental formation, various lactogenic hormones secreted by placental trophoblast cells replace pituitary-derived Prl to support CL function until the final parturition [6]. Apart from pituitary Prl and trophoblast lactogens, decidual cells also release factors belonging to the Prl family [7]. Upon embryo implantation in mice, uterine stromal cells experience decidualization and express decidual-specific Prl family members, e.g. Prl8a2 and Prl3c1 [8,9], which have been speculated contributive to CL maintenance and P4 production [10]. However, the functional relationship between pituitary Prl and these decidual-derived lactogens during pregnancy recognition and maintenance remains unclear.

Prl exerts its effects through the Prl receptor (Prlr) expressed in CL cells and activates downstream Stat5 [11,12]. The Prl signaling directly regulates genes related to P4 synthesis [13]. Mice with ablation of *Prl* or *Prlr* are infertile [14,15], while supplementation of exogenous P4 rescues early pregnancy failure in *Prlr*-deficient females [16,17], further reinforcing the essential role of Prl signaling in the maternal recognition of pregnancy in mice.

The Notch signaling is an evolutionarily conserved pathway that enables intercellular communication in short distance. Since its first discovery in Drosophila [18], the Notch pathway has been reported to affect a variety of fundamental biological aspects including cell proliferation, differentiation, death and fate decision [19]. In mammals, there are five ligands comprising three delta-like ligands (Dll1, Dll3, Dll4) and two jagged ligands (Jag1 and Jag2), all belonging to the Delta/Serrate/LAG-2 (DSL) family, and four receptors, Notch1-4 [20]. The ligand-receptor binding elicits sequential cleavages of the Notch receptor by gamma-secretases and subsequent release of the Notch intracellular domain (NICD) from the cell membrane [21,22]. The NICD then translocates into the nucleus, interacts with the CBF-1/Su(H)/LAG1 (CSL)/Rbpj transcription factor and recruits the transcriptional co-activator Mastermind-like (MAML) in place of transcriptional repressors to activate the transcription of target genes including the *Hes* and *Hey* family members [23,24].

Physiological roles of the Notch signaling in female reproduction have been widely investigated. All four Notch receptors as well as the Jagged1 and Dll4 ligand are expressed in the human endometrium [25], among which Notch1 modulates the progression of stromal-decidual transformation [26]. Similarly in mice, uterine-specific deletion of *Notch1* hampers decidualization by repressing cell cycle progression and triggering stromal cell apoptosis [27]. On the other hand, aberrant activation of the Notch1 pathway in the uterus leads to DNA hypermethylation of the progesterone receptor (PR) and thus compromises the P4 signaling and results in uterine developmental defects and infertility [28]. Notably, Notch proteins and ligands are expressed abundantly in endothelial cells and mural cells in the peri-implantation uterus [29], and Dll4 inhibition impairs angiogenesis during decidualization [30]. Meanwhile, our previous work has demonstrated the crucial role of Rbpj during early pregnancy. Specifically, Rbpj guides embryonic-uterine orientation and promotes decidual remodeling via Notch-independent and -dependent mechanisms, respectively [31].

In the present study, we intended to investigate the potential role of the Notch2 receptor in the uterus during peri-implantation period. Employing mice with uterine-specific deficiency of *Notch2*, we unraveled an unexpected involvement of uterine Notch2 in extending CL lifespan, guaranteeing P4 secretion and maintaining gestation via activating decidual Prl8a2 expression, and provided convincing proofs that decidual derived Prl family members contributed to pregnancy recognition and maintenance together with pituitary Prl.

## Results

### *Notch2* was efficiently ablated in the uterus

**Fig 1:**
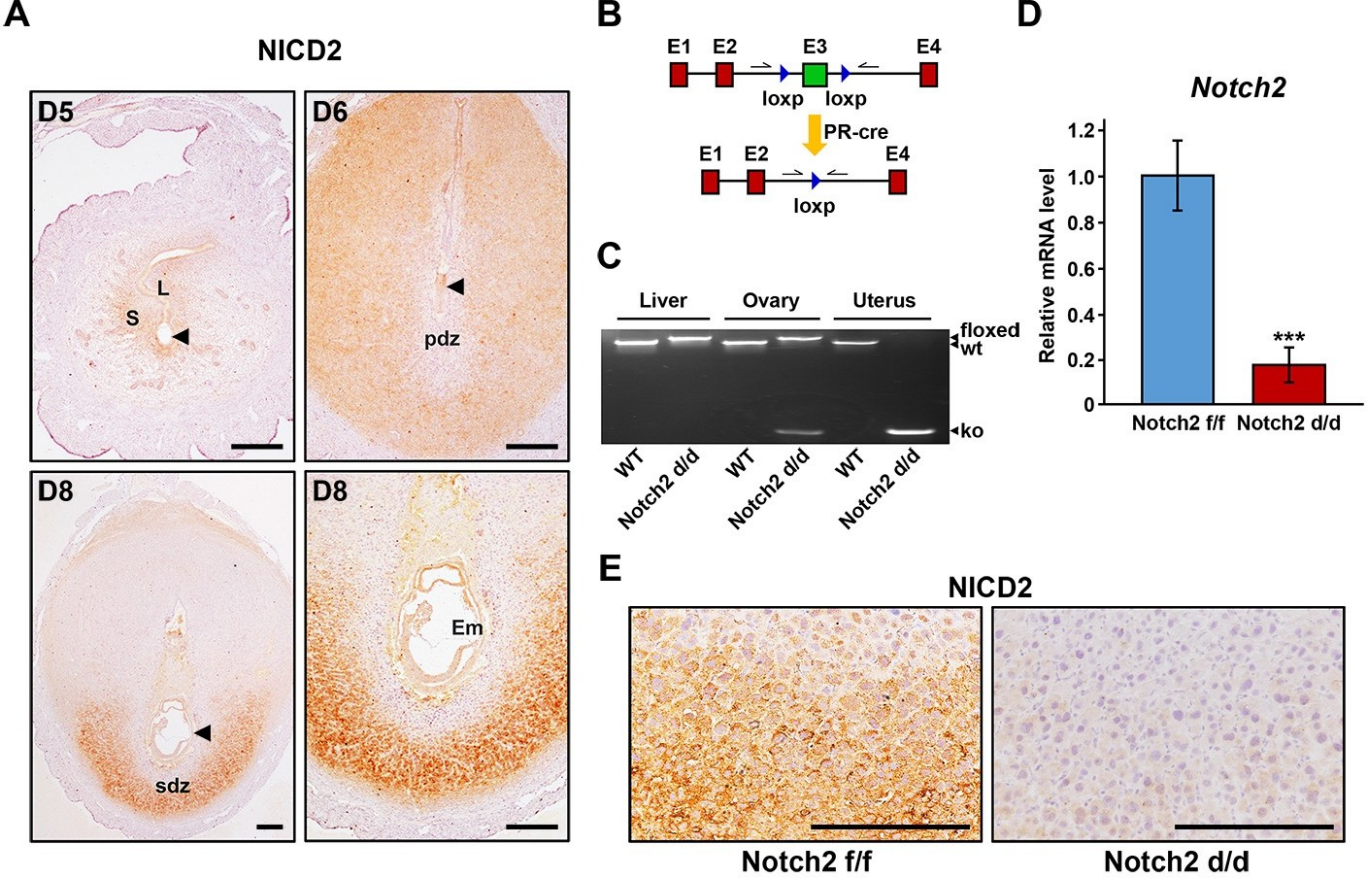
*Notch2* was efficiently deleted in the uterus. (A) The spatiotemporal expression pattern of NICD2 in the uterus during early pregnancy was revealed by immunohistochemistry. Arrowheads indicate the developing embryo. L, luminal epithelium; S, stroma; pdz, primary decidual zone; sdz, secondary decidual zone; Em, embryo. Scale bar: 100μm. (B) The scheme shows the strategy of uterine-conditional *Notch2* ablation. Arrows indicate primers used for the examination of knockout efficiency. (C) Genotyping analysis was carried out to validate knockout efficiency at DNA level in the liver, ovary and uterus. (D) Knockout efficiency at mRNA level in the uterus was further confirmed by QRT-PCR. Data are presented as mean±SEM. (E) Immunohistochemistry staining of NICD2 revealed that *Notch2* was efficiently ablated at protein level in the uterus. Scale bar: 100μm. ***p<0.001.

We first examined the spatiotemporal expression pattern of active Notch2 (NICD2) in the uterus during early pregnancy. Upon embryo implantation on day 5 of pregnancy, NICD2 appeared in epithelial and stromal cells surrounding the blastocyst. With the progression of decidualization from day 6 to day 8, the expression of NICD2 became more extensive throughout the secondary decidual zone (SDZ) rather than the primary decidual zone (PDZ) (Fig 1A). The localization of NICD2 was highly consistent with that of Rbpj [31], implying a potential role of Notch2-mediated canonical Notch signaling in early pregnancy events.

Considering that systemic *Notch2* knockout results in embryonic lethality [32], we introduced transgenic mice with Notch2 exon3 flanked by loxp sites (hereafter referred to as *Notch2 f/f*), and crossed them with the PR-driven cre recombinase (*PR-cre*) mouse line to specifically delete Notch2 in the uterus (hereafter referred to as *Notch2 d/d*) (Fig 1B). According to genotyping analysis (Fig 1C), quantitative real-time polymerase chain reaction (QRT-PCR) (Fig 1D) and immunohistochemistry (IHC) (Fig 1E), the knockout efficiency in the uterus was confirmed at DNA, mRNA and protein level, respectively. Notably, the ovary exhibited partial cre activity (Fig 1C) due to PR expression in the CL [33]. However, since Notch2 was not detected in the CL (S1A Fig), the level of NICD2 protein in the ovary was not affected despite the deletion of *Notch2* in the CL at DNA level.

### Uterine *Notch2* deficiency exerted no obvious influence on fertility

**Fig 2:**
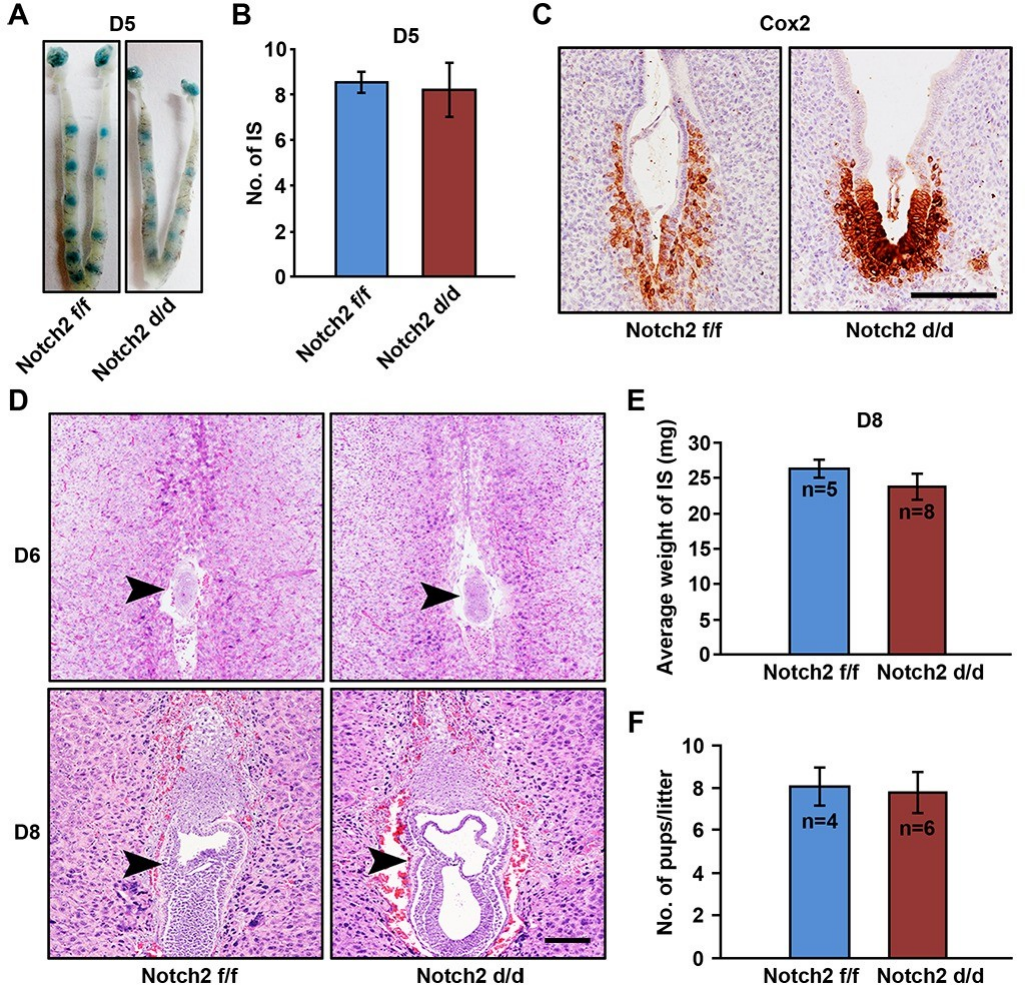
Uterine *Notch2* deficiency did not affect implantation, decidualization and fertility. (A) Implantation sites were visualized by intravenous injection of blue dye on day 5 in *Notch2 f/f* and *Notch2 d/d* mice. (B) The number of implantation sites on day 5 was comparable between *Notch2 f/f* and *Notch2 d/d* mice. IS, implantation sites. (C) Embryo attachment was detected on day 5 in *Notch2 f/f* and *Notch2 d/d* mice by immunohistochemistry staining for Cox2. Scale bar: 50μm. (D) H&E staining displayed normal embryo development on day 6 and 8 in *Notch2 f/f* and *Notch2 d/d* females. Arrowheads indicate the embryo. Scale bar: 50μm. (E) The average weight of implantation sites was measured on day 8 in *Notch2 f/f* and *Notch2 d/d* groups. (F) The litter size was compared between *Notch2 f/f* and *Notch2 d/d* females. Data in (B), (E) and (F) are presented as mean±SEM.

In order to uncover the involvement of Notch2 in early pregnancy events, we surveyed pregnancy status of *Notch2 f/f* and *Notch2 d/d* females at different timepoints. On day 5, embryo implantation occurred normally in both *Notch2 f/f* and *Notch2 d/d* mice, visualized by the blue dye reaction (Fig 2A), and the number of implantation sites (IS) was comparable (Fig 2B). In addition, Cox2 staining, a marker for embryo attachment [34], revealed successful implantation in *Notch2 d/d* mice (Fig 2C). In response to implantation, uterine stromal cells underwent decidual transformation to support embryonic development before placentation. Here, we also observed proper embryo development upon *Notch2* deficiency on day 6 and day 8 (Fig 2D). The average weight of IS in *Notch2 d/d* group rivaled that of *Notch2 f/f* group on day 8 (Fig 2E), suggesting no obvious defects during the period of decidualization. Furthermore, we compared the litter size between *Notch2 f/f* and *Notch2 d/d* mice, and found that uterine ablation of *Notch2* did not affect female fertility (Fig 2F).

### *Notch2 d/d* females were more sensitive to lipopolysaccharide (LPS)-induced Prl resistance

**Fig 3:**
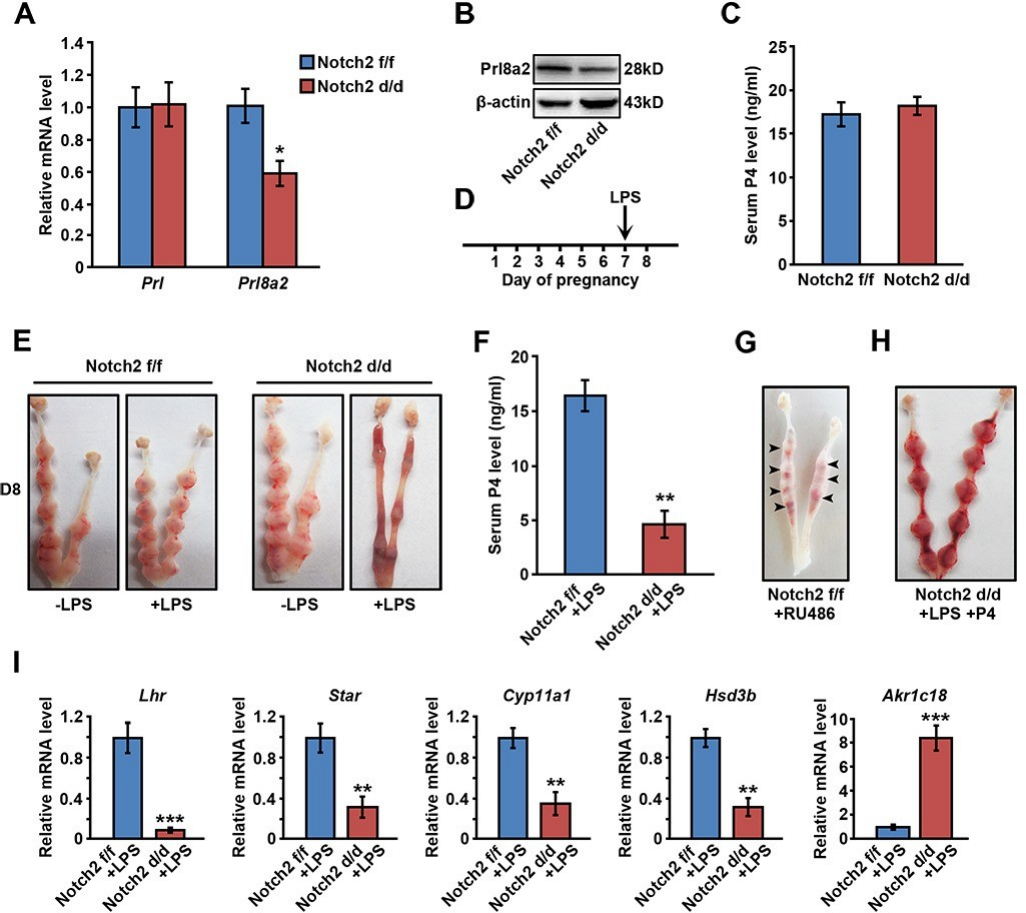
*Notch2 d/d* females were less tolerant to LPS-induced Prl resistance. (A) The relative mRNA level of *Prl* and *Prl8a2* in *Notch2 f/f* and *Notch2 d/d* decidua was examined by QRT-PCR. (B) Western blot analysis revealed the protein level of Prl8a2 in *Notch2 f/f* and *Notch2 d/d* decidua. (C) *Notch2 f/f* and *Notch2 d/d* females exhibited similar serum P4 level. (D) The scheme shows the LPS treatment schedule. (E) *Notch2 d/d* mice treated with low-dose LPS experienced pregnancy loss on day 8. (F) The serum P4 level was measured in LPS-treated *Notch2 f/f* and *Notch2 d/d* females. (G) RU486 injection resulted in abortion in *Notch2 f/f* mice. (H) Pregnancy failure in LPS-treated *Notch2 d/d females* was rescued by exogenous P4 supplementation. (I) The relative mRNA level of genes related to P4 synthesis or degradation was detected by QRT-PCR in ovaries of LPS-treated *Notch2 f/f* and *Notch2 d/d* females. Data in (A), (C), (F) and (I) represents mean±SEM. *p<0.5, **p<0.01, ***p<0.001.

Despite the fact that uterine *Notch2* deficiency did not hamper implantation and decidualization, we indeed noticed that the expression of Prl8a2, a member belonging to the Prl family that was specifically produced by decidual cells, was significantly decreased in the *Notch2 d/d* uterus at both mRNA and protein level (Fig 3A and 3B). Meanwhile, the level of Prl per se remained unchanged (Fig 3A). Decidual Prl family members were speculated involved in sustaining P4 production during pregnancy together with pituitary Prl [10]. Oil-induced decidualization in pseudopregnant mice led to elevated serum P4 level (S2A Fig), and treating cultured CL cells with the supernatant of decidualized uterus promoted P4 production (S2B Fig). We measured the level of serum P4 in *Notch2 f/f* and *Notch2 d/d* females, but did not find a significant difference between two groups (Fig 3C), possibly because pituitary derived Prl was sufficient to support CL function in *Notch2 d/d* mice. Notably, the expression of Prlr in CL was comparable between *Notch2 f/f* and *Notch2 d/d* mice (S1B Fig). We therefore attempted to interfere the Prl effect to investigate the contribution of decidual-specific Prl members to pregnancy maintenance.

It has been reported that LPS stimuli induces the ovarian resistance to Prl and therefore results in abnormal CL function, inadequate P4 production and pregnancy failure [35]. Here, we established a slight Prl resistance model by administrating low-dose LPS on day 7 (Fig 3D). In the *Notch2 f/f* group, pregnancy was not compromised on day 8, suggesting that *Notch2 f/f* females were tolerant to slight Prl resistance. However, *Notch2* deficiency led to early pregnancy loss in response to low-dose LPS treatment (Fig 3E). In the subsequent analysis, we unraveled that serum P4 level in *Notch2 d/d* mice was declined after LPS stimulation (Fig 3F), while exogenous P4 supplementation rescued abortion in LPS-treated *Notch2 d/d* mice (Fig 3H). Meanwhile, treatment of RU486, a progesterone receptor antagonist, gave rise to similar miscarriage in *Notch2 f/f* mice (Fig 3G), further indicating that the pregnancy failure of *Notch2 d/d* females in response to LPS was attributed to insufficient P4 secretion. In addition, we examined the expression of genes associated with P4 synthesis and degradation in ovaries. P4 synthesis related genes including *Lhr*, *Star*, *Cyp11a1* and *Hsd3b* were dramatically downregulated in *Notch2 d/d* ovaries, while the level of P4 degradative gene *Akr1c18* was significantly increased (Fig 3I). These findings suggested that females with uterine *Notch2* ablation showed less tolerance to LPS-induced Prl resistance due to abnormal CL function and insufficient P4 supply.

### *Notch2 d/d* females suffered from pregnancy failure when pituitary Prl signaling was blocked

**Fig 4:**
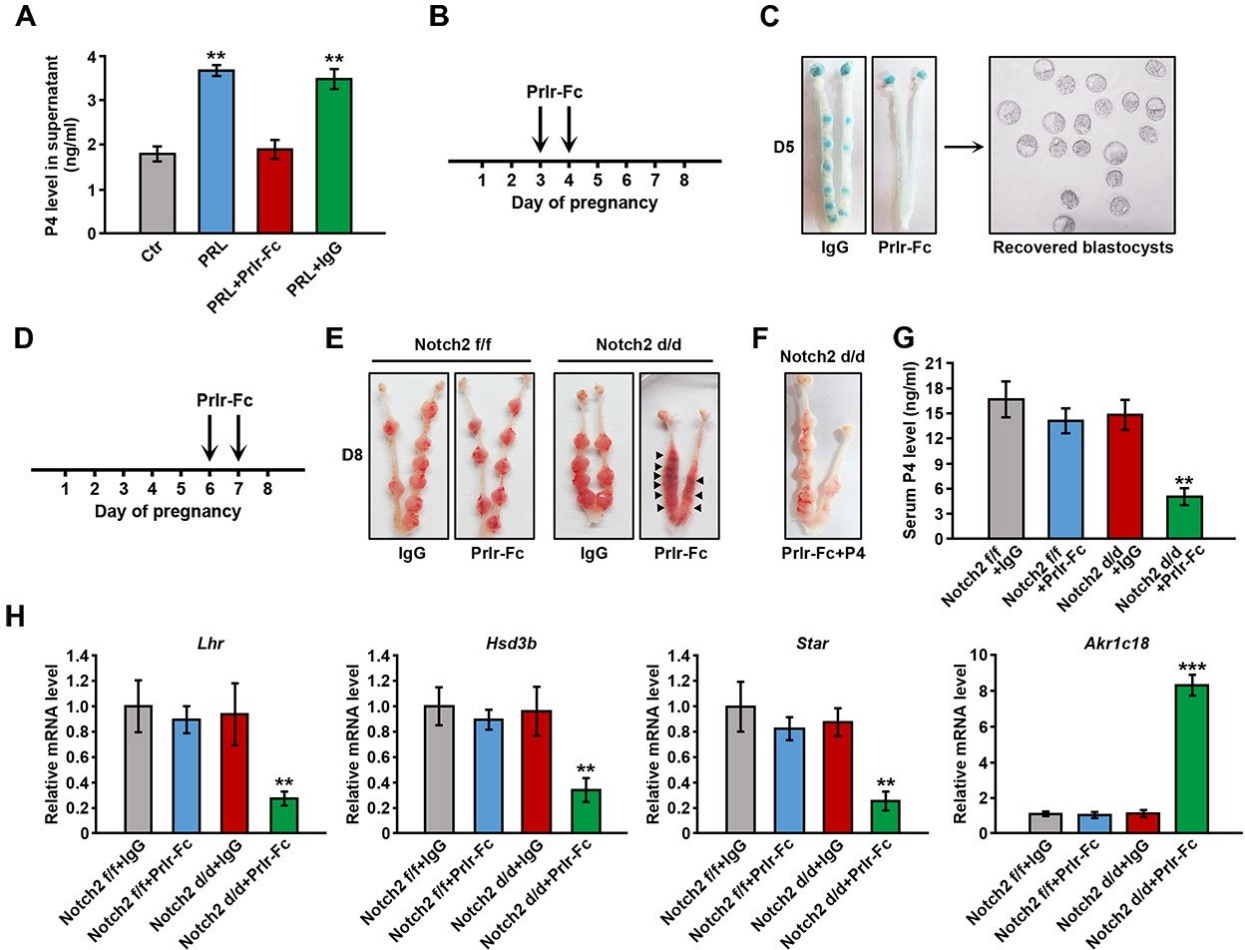
*Notch2 d/d* females ended up in abortion when Prl signaling was blocked. (A) P4 level in the supernatant of cultured CL cells was measured upon PRL, PRL+Prlr-Fc or PRL+IgG treatment. (B) The scheme shows the schedule of Prlr-Fc treatment. (C) Females treated with Prlr-Fc failed in implantation on day 5 despite the presence of morphologically normal blastocysts. (D) The schedule of Prlr-Fc treatment is shown. (E) *Notch2 d/d* females with Prlr-Fc administration suffered from pregnancy loss. Arrowheads indicate absorbed implantation sites. (F) Exogenous P4 supplementation alleviated abortion in *Notch2 d/d* females with Prlr-Fc administration. (G) The serum P4 level was measured in *Notch2 f/f* and *Notch2 d/d* females after Prlr-Fc treatment. (H) The relative mRNA level of genes related to P4 synthesis or degradation was examined by QRT-PCR analysis in ovaries of *Notch2 f/f* and *Notch2 d/d* females with Prlr-Fc administration. Mice with IgG administration were used as control. Data in (A), (G) and (H) represents mean±SEM. **p<0.01, ***p<0.001.

Decidual produced Prl family members differ greatly from pituitary Prl in structure, and do not share the canonical Prlr. We therefore blocked the pituitary Prl signaling using the Prlr-Fc fusion protein to solely explore the contribution of decidual Prl members. The Prlr-Fc fusion protein comprised the ligand-binding extracellular domain of Prlr and the Fc fragment of IgG, and could compete with Prlr for ligand binding without transmitting downstream signaling. We first validated the efficiency of Prlr-Fc in the in vitro CL culture system. Prl stimulated the production of P4 by CL cells as expected, which was effectively reversed by the addition of Prlr-Fc (Fig 4A). Furthermore, we tested the effectiveness of Prlr-Fc in vivo. P4 secretion by CL starting from day 3 of pregnancy, which depended on the Prl signaling, was essential to uterine receptivity and embryo implantation. We treated pregnant mice with Prlr-Fc on day 3 and 4, and then checked embryo implantation on day 5 (Fig 4B). After Prlr-Fc administration, females failed in implantation despite the presence of well-developed blastocysts (Fig 4C), implying that the Prlr-Fc fusion protein could successfully interrupt the Prl signaling in vivo.

Confirming the efficiency of the Prlr-Fc fusion protein, we next treated *Notch2 f/f* and *Notch2 d/d* females with Prlr-Fc on day 6 and day 7, and then examined the uterus on day 8 (Fig 4D). Prlr-Fc injection led to severe abortion in *Notch2 d/d* mice, but did not exerted obviously adverse effects on *Notch2 f/f* females (Fig 4E). Supplementation of exogenous P4 could rescue the pregnancy failure in Prlr-Fc treated *Notch2 d/d* mice (Fig 4F). In addition, serum P4 level was significantly decreased in *Notch2 d/d* mice after Prlr-Fc treatment (Fig 4G). Consistently, P4 synthesis-related genes were downregulated, while P4 degradation-related gene was upregulated (Fig 4H).

Pituitary release of Prl is precisely regulated by the dopamine system. The activation of the dopamine signaling inhibits Prl secretion [36], which was further proven by the phenomenon that dopamine receptor agonist bromocriptine-pretreated females exhibited compromised embryo implantation (S3A Fig). In the subsequent study, we repressed pituitary Prl release by bromocriptine to investigate the luteotropic role of decidual Prl members. Similar with Prlr-Fc, bromocriptine treatment resulted in decreased weight of IS on day 8 and declined serum P4 level (S3B, S3C and S3D Fig). These findings indicated that mice with uterine *Notch2* deficiency ended up in pregnancy failure in response to aberrant pituitary Prl signaling, owing to inadequate P4 production.

### Prl8a2 overexpression rescued LPS-induced abortion in *Notch2 d/d* mice by promoting P4 production

**Fig 5:**
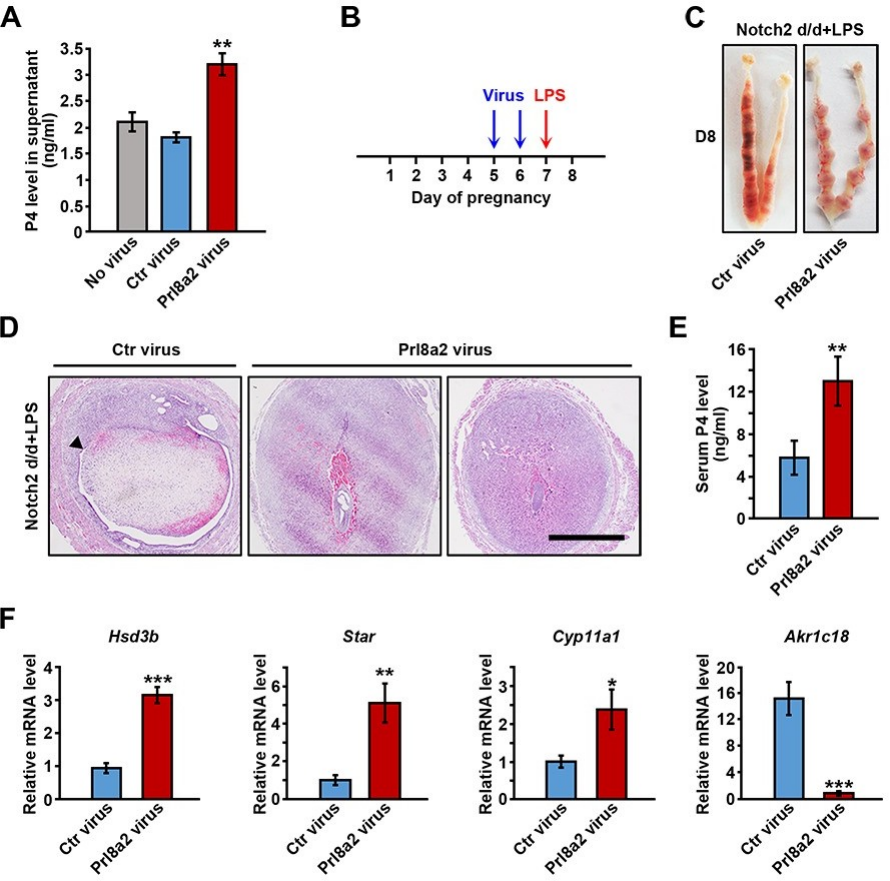
Prl8a2 overexpression rescued pregnancy failure in LPS-treated *Notch2 d/d* mice. (A) Lentivirus-mediated Prl8a2 overexpression prompted P4 secretion by cultured CL cells. (B) Virus injection and LPS treatment was carried out as illustrated. (C) Overexpression of Prl8a2 in *Notch2 d/d* mice rescued LPS-induced pregnancy failure. (D) H&E staining revealed normal embryo development in most implantation sites of LPS-treated *Notch2 d/d* mice after Prl8a2 overexpression. Arrowhead indicates abortion-induced decidual shedding. Scale bar: 300μm. (E) The serum P4 level was compared in LPS-treated *Notch2 d/d* mice with or without Prl8a2 overexpression. (F) The relative mRNA level of genes related to P4 synthesis or degradation in ovaries of LPS-treated *Notch2 d/d* mice with or without Prl8a2 overexpression. Data in (A), (E) and (F) are presented as mean±SEM. *p<0.5, **p<0.01, ***p<0.001.

Since the expression of decidual Prl8a2 was suppressed upon *Notch2* ablation, we wondered whether overexpression of Prl8a2 could rescue the abovementioned defect in the Prl resistance model. We implemented lentivirus-mediated Prl8a2 overexpression (S4A and S4B Fig). Prl8a2 overexpression remarkably augmented P4 secretion by cultured CL cells (Fig 5A). We then intravenously injected the virus into *Notch2 d/d* mice on day 5 and 6, followed by low-dose LPS treatment on day 7, and checked the uterus on day 8 (Fig 5B). Females pre-treated with the control virus ended up in abortion after LPS stimulation, while mice with Prl8a2 overexpression exhibited normal embryo development in most cases, except for embryo retardation in several IS (Fig 5C and 5D). In addition, Prl8a2 overexpression resulted in elevated serum P4 level (Fig 5E), upregulation of P4 synthesis-related genes as well as downregulation of P4 degradation-associated gene (Fig 5F). These findings provided convincing evidence that decidual Prl8a2 contributed to the maintenance of CL function during post-implantation period in mice.

### Decidual Prl8a2 was modulated by the canonical Notch signaling pathway

**Fig 6:**
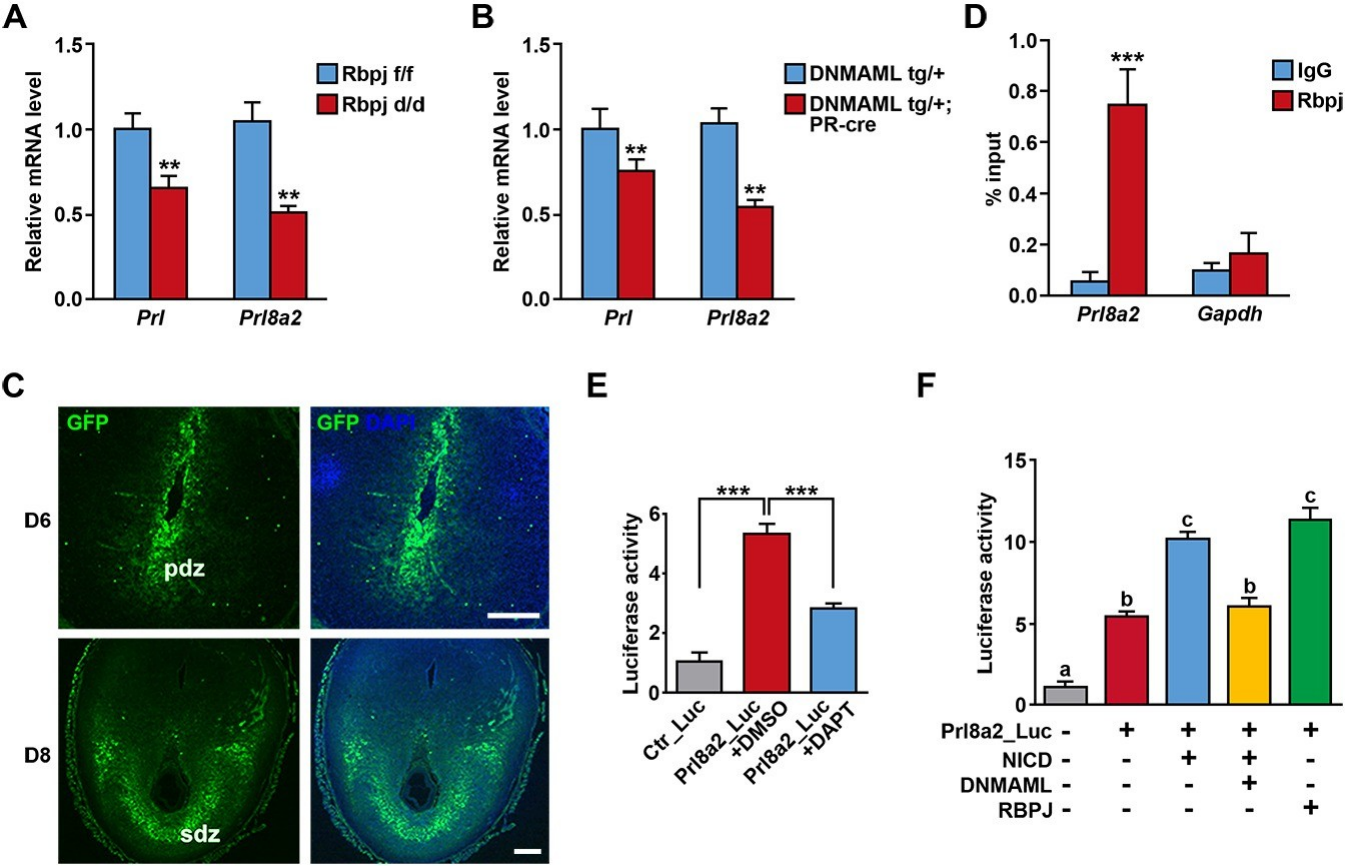
Prl8a2 was under the control of canonical Notch signaling. The relative mRNA level of *Prl* and *Prl8a2* was examined in *Rbpj f/f* versus *Rbpj d/d* decidua (A) and *DNMAML tg/+* versus *DNMAML tg/+;PR-cre* decidua (B). (C) GFP signals in implantation sites of mice carrying Rbpj responsive element-driven GFP on day 6 and 8. Nuclei were stained by DAPI. pdz, primary decidual zone; sdz, secondary decidual zone. Scale bar: 50μm and 100μm. (D) Chromatin immunoprecipitation assay showed the direct binding of Rbpj on Prl8a2 promoter. IgG was served as isotype control. *Gapdh* was served as negative control. (E) Luciferase analysis revealed Prl8a2 promoter activity in the presence of DAPT or DMSO. (F) Prl8a2 promoter activity was enhanced by NICD and RBPJ, which was reversed by DNMAML. Data in (A), (B) (D) and (E) are presented as mean±SEM. **p<0.01, ***p<0.001, a vs b and b vs c: P < 0.01, a vs c: P < 0.001.

Given that uterine *Notch2* deficiency led to downregulation of decidual Prl8a2, we next investigated the possibility that Prl8a2 was regulated by the canonical Notch pathway. We adopted two mouse lines with either deletion of *Rbpj* or overexpression of DNMAML in the uterus. DNMAML only contains the N terminus of MAML, which is capable of binding NICD without recruiting transcriptional co-activators and thus interferes endogenous MAML function [37]. In both mouse models, the expression level of decidual *Prl* and *Prl8a2* was significantly declined (Fig 6A and 6B), implying that decidual Prl members might be under the control of the canonical Notch pathway. Meanwhile, employing mouse model carrying green fluorescent protein (GFP) reporter driven by Rbpj responsive element, we observed activation of the canonical Notch signaling in decidual cells (Fig 6C), in accord with the expression pattern of NICD2 (Fig 1A), further indicating the potential participation of canonical Notch pathway in decidual transformation and decidual Prl members expression.

We screened the promoter region of Prl8a2 and sought out a conserved Rbpj binding sequence 3000bp upstream the transcription start site. Chromatin immunoprecipitation (ChIP) assay demonstrated the direct binding of Rbpj at this site (Fig 6D). Furthermore, we cloned this site into the luciferase reporter system, and found that DAPT, which inhibited the gamma-secretase complex and the release of NICD, could obviously repress Prl8a2 promoter activity (Fig 6E). In the subsequent analysis, we revealed that NICD could remarkably activate the Prl8a2 promoter, which was reversed by simultaneous overexpression of DNMAML. In addition, Rbpj alone significantly enhanced the activity of Prl8a2 promoter (Fig 6F). These findings further confirmed the direct regulation of Prl8a2 transcription by canonical Notch signaling pathway.

## Discussion

**Fig 7:**
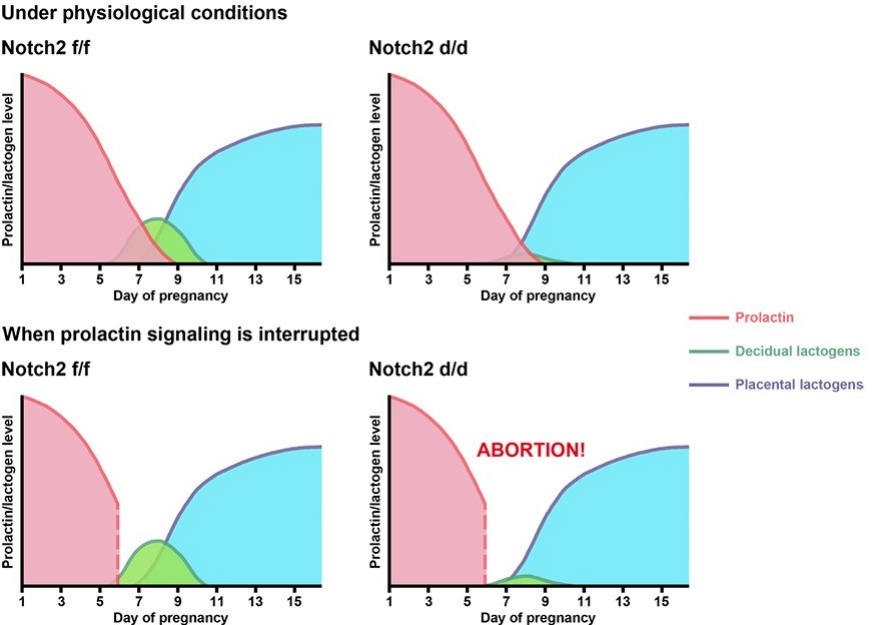
Working model. Under normal physiological circumstances, pituitary prolactin sustains CL function until mid-gestation, which is then taken over by placental lactogens. Decidual lactogens assist pituitary prolactin in pregnancy maintenance, while the latter plays the predominant role. Therefore, no obvious defects in fertility were noticed in *Notch2 d/d* mice. However, when the prolactin signaling is blocked, decidual lactogens were capable of ensuring progesterone production in *Notch2 f/f* mice, but *Notch2 d/d* mice experienced pregnancy loss due to insufficient level of decidual lactogens.

The establishment and maintenance of gestation necessitates not only the intimate interactions between the conceptus and the uterus, but also the adaptive changes in other maternal systems and organs. Sexual intercourse ignites a neuroendocrine reflex, prompting Prl surges released from the anterior pituitary, which extends the CL lifespan and guarantees P4 provision during early pregnancy [4]. In the present study, we uncovered an unexpected role of uterine Notch2 in pregnancy maintenance via upregulating decidual Prl8a2, a member belonging to the Prl family, and provided explicit evidence that decidual Prl8a2 facilitated CL maintenance and P4 production along with Prl released from the pituitary gland (Fig 7).

In mammals, embryonic development takes place in the maternal uterus. In response to blastocyst implantation, the endometrium experiences stromal-decidual transformation to provide an appropriate environment for the growing conceptus [38,39]. Plentiful researches have been carried out regarding the involvement of Notch signaling in the uterus during early pregnancy [27–29,40–42]. In our previous study, we uncovered dual roles of Rbpj, the nuclear transducer of the Notch signaling pathway, in blastocyst orientation and stromal decidualization via Notch-independent and -dependent mechanisms, respectively [31]. Considering the Notch-dependent participation of Rbpj during the period of decidual remodeling, we attempted to excavate the upstream Notch receptor that might be involved. We screened several Notch receptors and eventually put emphasis on Notch2, since its spatiotemporal expression pattern was highly consistent with that of Rbpj particularly during decidualization (Fig 1A), and its role in early pregnancy events had not been explored. Owing to embryonic lethality resulting from systemic *Notch2* deficiency [32], we specifically deleted *Notch2* in the uterus by employing the *PR-Cre* mouse model. To our surprise, we did not find obvious defects in female fertility upon *Notch2* ablation (Fig 2), which might be attributed to the compensatory effect by other Notch receptors.

In the further detailed analysis, we noticed that the expression of Prl family members, e.g. Prl8a2, was decreased in the *Notch2 d/d* decidua. The physiological roles of these decidual Prl members were unclear. It had been hypothesized that decidual derived Prl family members share similar function with the pituitary Prl in pregnancy recognition and maintenance [10]. In *Notch2 d/d* females, the CL production of P4 and pregnancy maintenance were not compromised despite the declined level of decidual Prl members. We speculated that this might be due to the predominant function of Prl released from the pituitary gland. Therefore, several models were established to weaken the leading role of pituitary Prl, including the LPS-induced Prl resistance model, blocking the Prl signaling using the Prlr-Fc fusion protein, as well as inhibiting pituitary Prl release using the dopamine receptor agonist bromocriptine.

However, it is hard to deny that each model has its limitations. In the LPS-induced Prl resistance model, although low-dose LPS indeed enforced the ovarian resistance to the Prl signaling and impaired pregnancy in *Notch2 d/d* mice (Fig 3), these defects might also arose from the inflammatory reaction in the endometrium elicited by LPS, since LPS-induced endometrial inflammation is detrimental to embryo survival [43–47]. In the case of interrupting Prl signaling with Prlr-Fc, the Prlr-Fc fusion protein efficiently blocked the Prl signaling and disturbed CL function (Fig 4). However, Prlr is expressed in the uterus as well as the developing embryo in addition to the CL [17], implying the existence of other targets of the Prl signaling. As a matter of fact, Prl could also target the uterus and repress the production of factors that were harmful to gestation maintenance [48]. Therefore, the pregnancy loss in Prlr-Fc treated *Notch2 d/d* females might also be attributed to the dysregulated Prl signaling in the uterus. In bromocriptine-treated *Notch2 d/d* mice, the phenotype was not as severe as that in the other two models (S3 Fig), which might be owing to the method of drug delivery. The release of Prl form the pituitary is fluctuant, so the timing of bromocriptine administration might directly influence the consequences [49]. In spite of the respective flaw in each model, the combination of these three models provided convincing evidence that decidual Prl family members facilitated pituitary Prl to sustain the CL function and P4 secretion during gestation.

Our study highlighted the importance of decidual Prl8a2 in supporting CL function. Prl8a2 exhibits abundant and specific expression in decidualized stromal cells [50–52], and thus serves as a widely-accepted marker for decidualization, but little is known with respect to its physiological functions. In the present study, our in vivo and in vitro experiments confirmed that Prl8a2 promoted P4 secretion from the CL (Fig 5), which was the first time to our knowledge that the luteotropic role of Prl8a2 has been demonstrated. It has been reported that *Prl8a2* knockout female mice displayed no obvious abnormality during pregnancy under normal circumstances, which consisted with the phenotype of *Notch2 d/d* mice, but were more sensitive to hypoxia stimuli [53]. However, it is unclear whether this was attributed to insufficient CL function and P4 secretion. Given that Prl8a2 does not share the same receptor with Prl, the specific receptor that mediates Prl8a2 effects warrants further identification.

In human, Prl is expressed abundantly by decidualized stromal cells. The decidual Prl gene possesses a different promoter from that of the pituitary Prl, which results from the insertion of transposon element during evolution [54]. This inserted region is enriched with binding sites for multiple transcription factors [55], implying a complicated regulatory mechanism. However, the molecular mechanism by which the expression of Prl8a2 is regulated remains elusive. We noticed that in mice with uterine-conditional deletion of *Rbpj* or overexpression of DNMAML, the expression of Prl8a2 displayed similar downregulation. In addition, a conserved Rbpj binding site was found upstream of the Prl8a2 promoter, and ChIP assay together with luciferase analysis confirmed that Prl8a2 expression was controlled by the canonical Notch pathway (Fig 6). Whether the expression of Prl8a2 is modulated by other signaling pathways or transcription factors requires more detailed investigations.

## Materials and methods

### Ethics statement

All mice used in the research were housed in the Animal Care Facility of Xiamen University, according to guidelines for the use and care of laboratory animals. All experimental procedures were approved by the Animal Welfare Committee of Research Organization (X200811), Xiamen University.

### Animals and treatments

*Notch2 f/f* mice were purchased from Jackson Laboratory. *Rbpj f/f* and *DNMAML tg/+* mice were generated as previously described [56,57]. Uterine-specific mutant mice were generated by crossing *Notch2 f/f* or *Rbpj f/f* or *DNMAML tg/+* mice with *PR-Cre* mice [33].

Female mice at the age of 6–8 weeks were mated with fertile males to induce pregnancy (day 1 represented the day when vaginal plug was seen). Pregnant females were sacrificed at different time points. On day 5 of pregnancy, implantation sites were visualized by intravenous injection of 100μl Chicago blue (Sigma, C8679), and the number of implantation sites was recorded. On day8, the weight of implantation sites was recorded. Implantation sites, ovaries and blood samples were collected.

For LPS stimulation, 1mg LPS (Sigma, L2880) dissolved in 100μl saline was intraperitoneally injected on day 7. For Prlr-Fc fusion protein treatment, 1mg Prlr-Fc (Provided by Ziqiang Li (Beijing VDJBio Co. LTD)) dissolved in 100μl phosphate buffered saline (PBS) was intraperitoneally injected on day 6 and 7. For bromocriptine administration, pregnant females received a gavage of 100μg bromocriptine mesylate (Sigma, Y0000677) on day 6 and 7. For exogenous progesterone supplementation, 2mg progesterone (Sigma, P0130) dissolved in 100μl sesame oil (Sigma, S3547) was subcutaneously injected. For RU486 administration, 300ug RU486 (Sigma, M8046) dissolved in 100μl PBS was intraperitoneally injected. For Prl8a2 overexpression, 0.1 ml concentrated lentivirus was intravenously injected at indicated time points.

For oil-induced pseudopregnancy model, females were mated with vasectomized males, and sesame oil was infused into the uterine lumen on day 4, to mimic blastocysts transferring into the uterus.

### P4 level measurement

Blood samples and CL cell culture supernatants were collected and the P4 level was measured by radioimmunoassay (RIA).

### Hematoxylin and eosin staining

Collected implantation sites were fixed in 10% neutral buffered formalin (NBF) overnight at room temperature (RT), dehydrated in ethanol and xylene, and then embedded in paraffin. 5μm paraffin sections were deparaffinized and rehydrated using xylene and ethanol. Hematoxylin and eosin staining was performed according to manufacturer’s instructions (Solarbio, G1120).

### Immunohistochemistry

Implantation site and ovary samples were fixed in 10% NBF overnight at RT, dehydrated using ethanol and xylene, and embedded in paraffin. 5μm paraffin sections were deparaffinized and rehydrated in xylene and ethanol. Rehydrated sections were then subjected to heat-mediated epitope retrieval for 15min at 120°C in sodium citrate buffer, treated with 3% hydrogen peroxide/methanol solution for 10min at RT. Antigen blocking was performed using 0.5% bovine serum albumin (BSA) for 1h at RT. Subsequently, sections were incubated with primary antibody overnight at 4°C, washed by phosphate buffered saline (PBS) for 3 times, and incubated with horse radish peroxidase (HRP)-conjuncted secondary antibody (NeoBioscience, ANR02-2, dilution: 1:200) for 1h at RT, and developing was performed using the diaminobenzidine (DAB) kit (ZSGB-BIO, ZLI-9018). Antibodies against NICD2 (Abcam, ab52302, dilution: 1:200) and Cox2 (Servicebio, GB11077-1, dilution: 1:200) were used in the research.

### In situ hybridization

For probe construction, target gene cDNAs were amplified by PCR, and digoxin (DIG)-labeled probes were generated by in vitro transcription using the DIG RNA labeling mix (Roche, 11277073910). Sequences of primers used for probe production were: 5’ ACTTGCCTGCCAAAGAGAGAA 3’ (forward) and 5’ TGACTGTCCATTCAGGAGGC 3’ (reverse). For in situ hybridization, frozen sections were fixed in 4% paraformaldehyde (PFA) for 1h at RT, treated with acetic anhydride-containing triethanolamine solution for 10min at RT, and then subjected to probe incubation overnight at 60°C. Subsequently, sections were treated with RNase for 30min at 37°C, and incubated with anti-DIG antibody (Roche, 11214667001, dilution: 1:3000) overnight at 4°C. Developing was performed using nitroblue tetrazolium (NBT, Gentihold, N1332) and 5-bromo-4-chloro-3-indolyl-phosphate (BCIP, Gentihold, B1360) after 10min levamisole treatment.

### RNA extraction and QRT-PCR

For RNA extraction, frozen tissues were ground in Trizol and subjected to chloroform extraction, isopropanol precipitation and ethanol washing. Reverse transcription and QRT-PCR were performed according to manufacturer’s instructions (Takara, RR047A & RR820A). Primer sequences used in QRT-PCR analysis were listed in S1 Table.

### Protein extraction and Western blot

For protein extraction, cells were lysed in ice-cold RIPA buffer for 1h and subjected to centrifugation (20000g, 30min, 4°C). Supernatant was collected. Protein samples were subjected to electrophoresis, electrotransfer, membrane blocking using 5% skim milk for 1h at RT and primary antibody incubation overnight at 4°C. Home-made rabbit polyclonal primary antibody against mouse Prl8a2 was used at a dilution of 1:1000. The membrane was washed by Tris buffered saline Tween (TBST) for 3 times, and incubated with HRP-conjuncted anti-rabbit secondary antibody (NeoBioscience, ANR02-2, dilution: 1:5000) for 1h at RT, and developing was performed using the SuperSignal West Pico PLUS Chemiluminescent Substrate (Thermo Fisher, 34578).

### Chromatin immunoprecipitation

Cultured decidualized stromal cells were crosslinked in 1% formaldehyde/PBS solution for 20min at RT, which was terminated by 0.125M glycine for 10min on ice. Cells were collected after PBS washing, and subjected to lysis and sonication until the chromatin was sheared to an average length of 200-400bp. 1% of chromatin fragments was served as input, while the rest was divided equally, and incubated with antibody against Rbpj (CST, 5313, dilution: 1:50) and IgG (CST, 3900, dilution: 1:50) overnight at 4°C. The DNA-protein-antibody complexes were incubated with protein A magnetic beads for 3-4h at 4°C. Beads together with the DNA-protein-antibody complexes were collected and washed in the order of low salt buffer (containing 150mM NaCl), high salt buffer (containing 500mM NaCl), LiCl buffer (containing 250mM LiCl) and TE buffer. Precipitated DNA-protein complexes were eluted from beads and subjected to crosslink reversal in 5M NaCl for 8h at 65°C. DNA was purified by phenol chloroform isoamyl alcohol extraction and ethanol precipitation. Primers used in the subsequent QRT analysis were designed targeting 3000bp upstream Prl8a2 transcription start site: 5’ CCTGTAGATAGATTGCTGGGGC 3’ (forward) and 5’ TAATCGCCTCCCAACAGACTTC 3’ (reverse).

### In vitro corpus luteum cell culture

Isolated ovaries from D4 pregnant females were placed in the DMEM-F12 medium (Sigma-Aldrich, D6434). CL was separated and digested by collagenase (Gibco, 17018–029) for 20min at 37°C. Digested CL cells were collected by centrifugation and cultured in DMEM-F12 supplemented with 10% charcoal-stripped fetal bovine serum (CS-FBS) (Biological Industries, 04-201-1A), 88μg/ml sodium pyruvate and 292μg/ml L-glutamine.

### Luciferase reporter analysis

The conserved Rbpj binding sequence upstream the transcription start site of Prl8a2 was cloned into the pGL3-Basic vector (Promega). The sequences of primers used in vector construction are listed in S2 Table. The pRL-TK plasmid (Promega) was used as internal control. Plasmids were transfected into 293T cells. Cells were collected 48h after transfection, and fluorescence was measured using the dual luciferase reporter system (Promega).

### Lentivirus vector construction and packaging

The coding sequence of Prl8a2 were amplified by PCR, and inserted into the pLVX-IRES-ZsGreen1 vector (Clontec). The sequences of primers used in vector construction are listed in S2 Table. The modified vector was transfected into 293T, and viruses were collected from the supernatant 3 days after transfection.

### Statistics

All data were presented as mean±S.E.M. Comparation between two groups was analyzed with t tests, while one-way ANOVA was performed for comparation between more than two groups. P<0.05 was considered statistically significant.

## Supporting information

S1 Fig Uterine Notch2 deficiency did not influence ovarian Notch2 and Prlr expression. (A) The expression of NICD2 was detected by immunohistochemistry in Notch2 f/f and Notch2 d/d ovaries. (B) In situ hybridization analysis revealed Prlr expression in Notch2 f/f and Notch2 d/d ovaries. CL, corpus luteum. Scale bar: 100 μm and 200 μm.
(TIF)

S2 Fig Decidua possessed luteotropic effects. (A) The serum P4 level in pseudopregnant females with or without oil-induced decidualization was measured at indicated time points. (B) The supernatant of decidualized uterus promoted P4 secretion by cultured CL cells compared to undecidualized uterus. Data represent mean±SEM. *p<0.5, **p<0.01.
(TIF)

S3 Fig Notch2 d/d females were more sensitive to bromocriptine-induced pituitary Prl blockage. (A) Females pretreated with bromocriptine exhibited impaired implantation despite the presence of morphologically normal blastocysts. (B) The size of implantation sites in Notch2 d/d mice pretreated with bromocriptine was smaller compared to Notch2 f/f mice, and the average weight of implantation sites was declined according to (C). (D) The serum P4 level was detected in Notch2 f/f and Notch2 d/d mice pretreated with bromocriptine. Data in (C) and (D) represent mean±SEM. **p<0.01.
(TIF)

S4 Fig Lentivirus-mediated Prl8a2 overexpression. (A) GFP signals in 293T cells transfected with Prl8a2 overexpression vectors. (B) Western blot analysis revealed Prl8a2 expression in 293T cells after transfection. NS, non-specific band.
(TIF)

S1 Table Primer sequence for QRT-PCR. (DOCX)

S2 Table Primer sequence for vector construction. (DOCX)
