# Supplementary figures and images for: Uterine Notch2 facilitates pregnancy recognition and corpus luteum maintenance via upregulating decidual Prl8a2
Source: PLoS Genet. 2021 Aug 30;17(8):e1009786. doi: 10.1371/journal.pgen.1009786 (PMC8432799; doi:10.1371/journal.pgen.1009786)

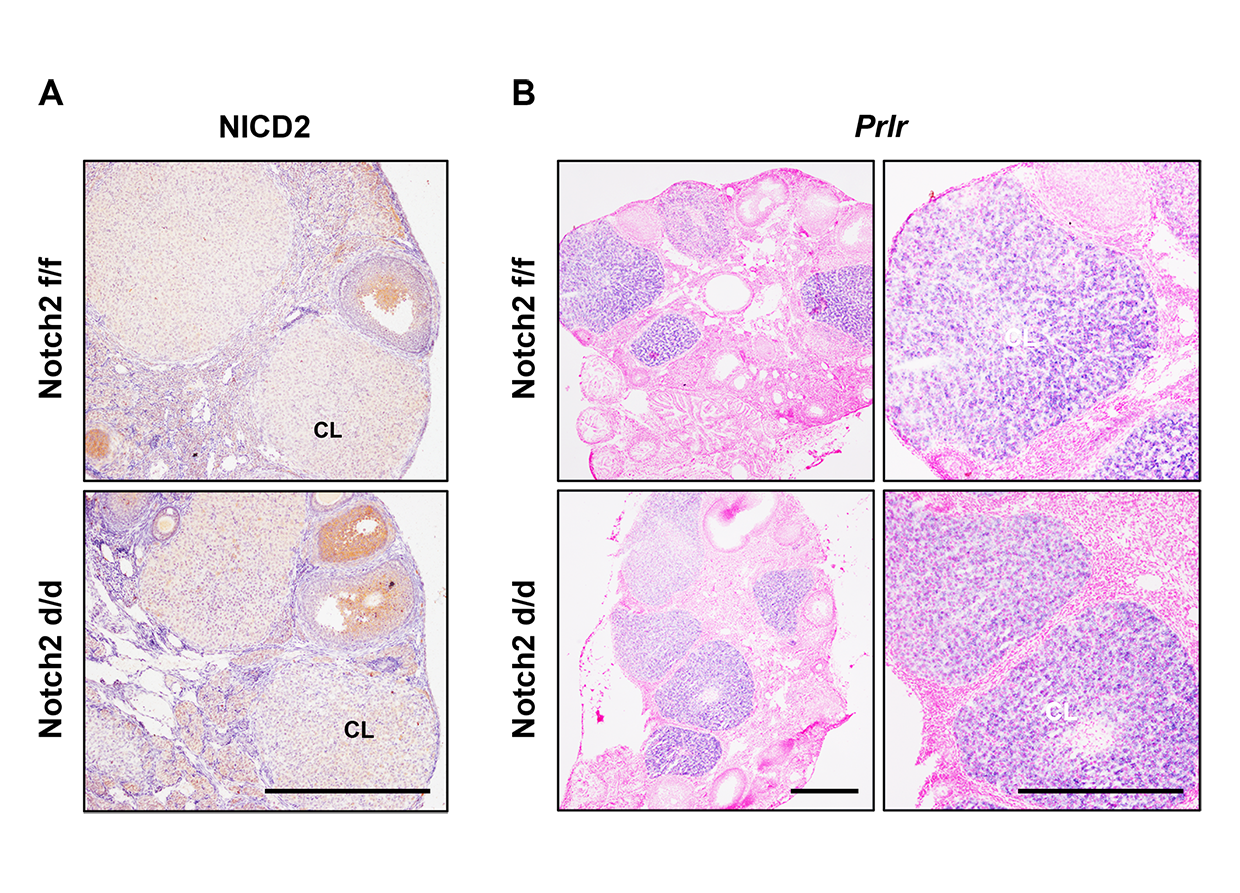

Supplement: S1 Fig — (A) The expression of NICD2 was detected by immunohistochemistry in Notch2 f/f and Notch2 d/d ovaries. (B) In situ hybridization analysis revealed Prlr expression in Notch2 f/f and Notch2 d/d ovaries. CL, corpus luteum. Scale bar: 100 μm and 200 μm. (TIF) [file pgen.1009786.s001.tif]

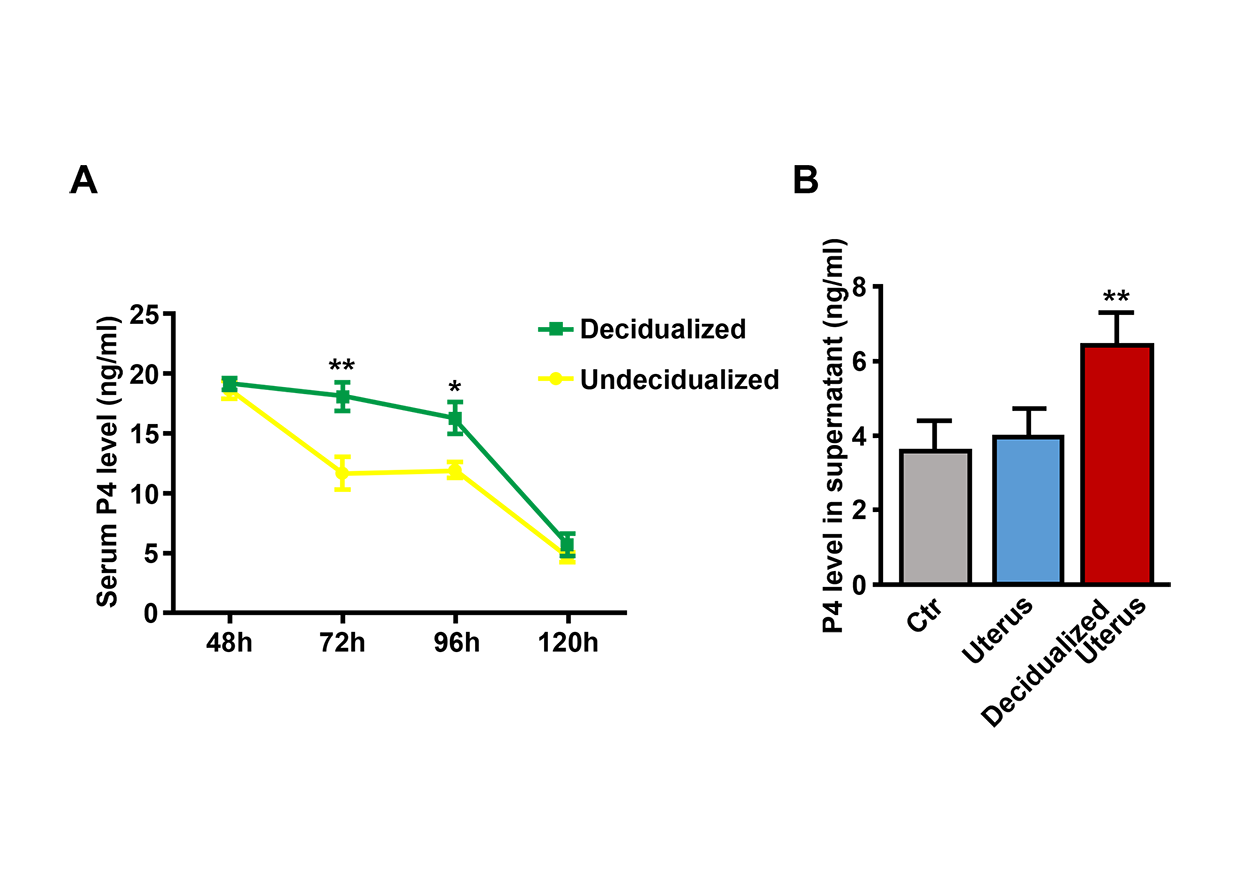

Supplement: S2 Fig — (A) The serum P4 level in pseudopregnant females with or without oil-induced decidualization was measured at indicated time points. (B) The supernatant of decidualized uterus promoted P4 secretion by cultured CL cells compared to undecidualized uterus. Data represent mean±SEM. *p<0.5, **p<0.01. (TIF) [file pgen.1009786.s002.tif]

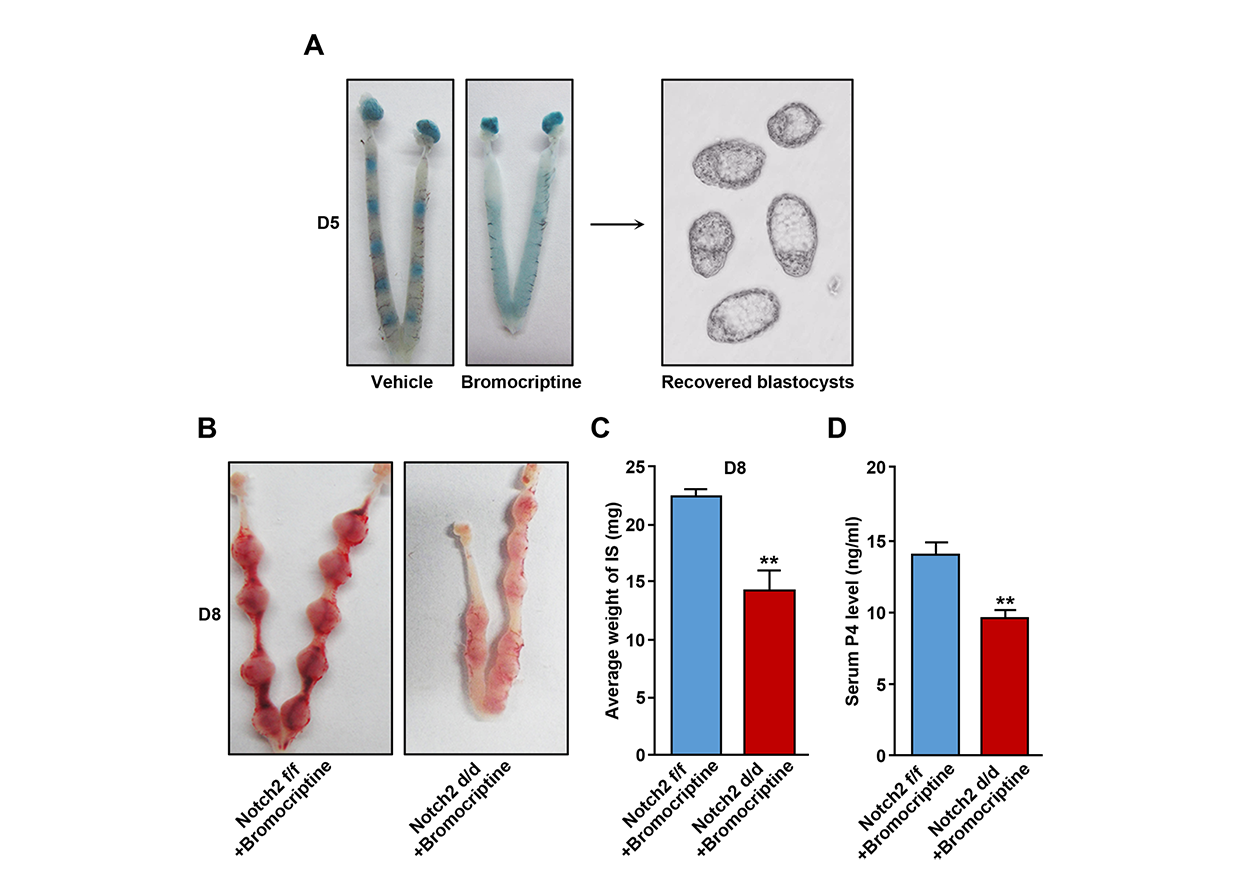

Supplement: S3 Fig — (A) Females pretreated with bromocriptine exhibited impaired implantation despite the presence of morphologically normal blastocysts. (B) The size of implantation sites in Notch2 d/d mice pretreated with bromocriptine was smaller compared to Notch2 f/f mice, and the average weight of implantation sites was declined according to (C). (D) The serum P4 level was detected in Notch2 f/f and Notch2 d/d mice pretreated with bromocriptine. Data in (C) and (D) represent mean±SEM. **p<0.01. (TIF) [file pgen.1009786.s003.tif]

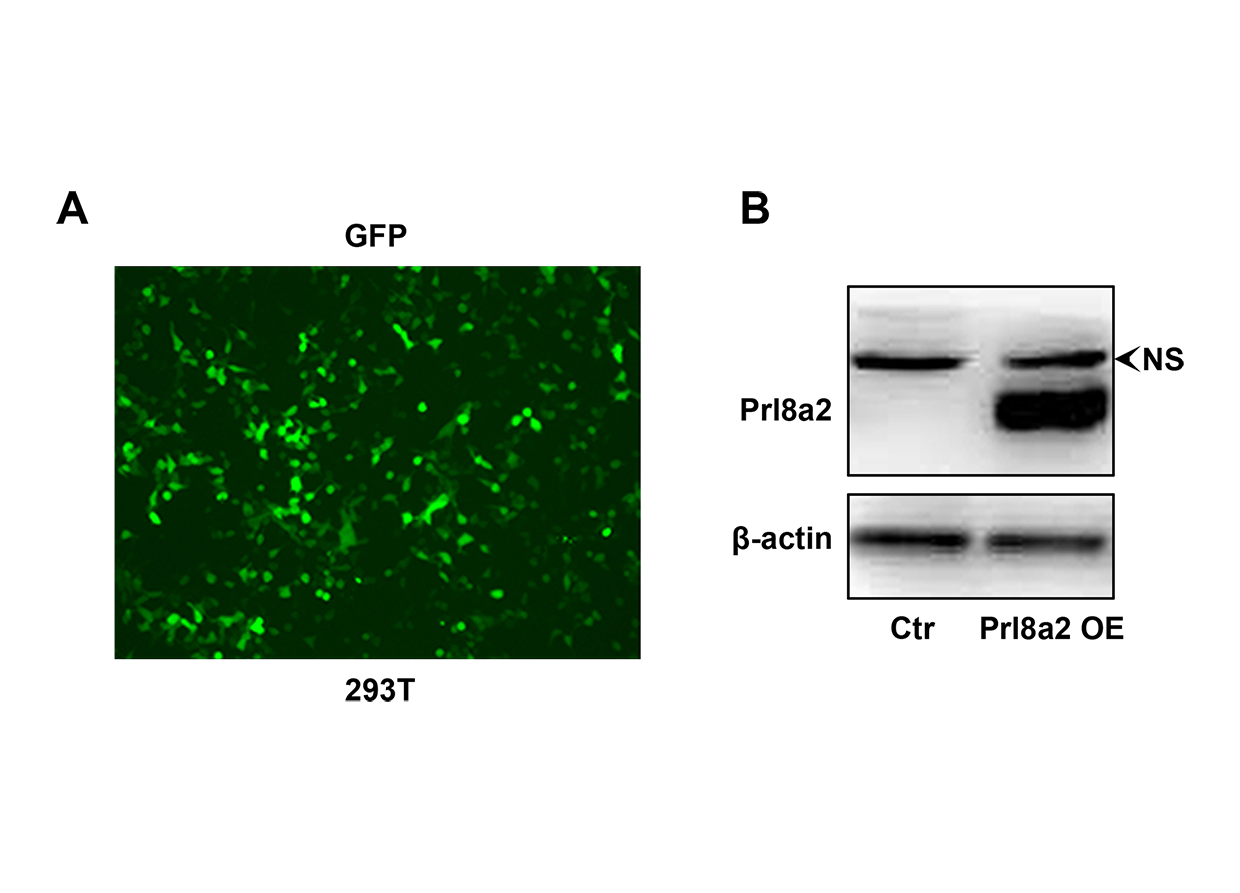

Supplement: S4 Fig — (A) GFP signals in 293T cells transfected with Prl8a2 overexpression vectors. (B) Western blot analysis revealed Prl8a2 expression in 293T cells after transfection. NS, non-specific band. (TIF) [file pgen.1009786.s004.tif]
