# Supplementary material for: Uterine Notch2 facilitates pregnancy recognition and corpus luteum maintenance via upregulating decidual Prl8a2
Source: PLoS Genet. 2021 Aug 30;17(8):e1009786. doi: 10.1371/journal.pgen.1009786 (PMC8432799; doi:10.1371/journal.pgen.1009786)
Supplement: S1 Table — (DOCX) [file pgen.1009786.s005.docx]

**S1 Table. Primer sequence for QRT-PCR.**

| Gene name | Primer sequence |
| --- | --- |
| *Notch2* | 5’-TATCGCCCAGACATTCTCGC-3’  5’-ACACACTGACGGGGATCAAC-3’ |
| *Prl* | 5’-GCAGGGATTCCCACAAGGTT-3’  5’-GAGGAGACCAATTGCACCCA-3’ |
| *Prl8a2* | 5’-AAGAATGCCCTTCAGCGAGC-3’  5’-AGCTGGTGGGTTTGTGACAT-3’ |
| *Lhr* | 5’-CAGGCCTGTGAGTGCAGATA-3’  5’-GTGCTCTCAGTTGGGTAGGC-3’ |
| *Star* | 5’-TCGCTACGTTCAAGCTGTGT-3’  5’-ACGTCGAACTTGACCCATCC-3’ |
| *Cyp11a1* | 5’-CCCATCCTCTTCAACTTCCA-3’  5’-AGAGGAGAGCCCAGCTAACC-3’ |
| *Hsd3b* | 5’-GCTTCCTGCTACGTCCAGTC-3’  5’-ATCTCCCTGTGCTGTTCCAC-3’ |
| *Akr1c18* | 5’-TAGGCCAGGCCATTCTAAGC-3’  5’-CTCCATGGCCTTCAGAGACAC-3’ |
