# Supplementary material for: Uterine Notch2 facilitates pregnancy recognition and corpus luteum maintenance via upregulating decidual Prl8a2
Source: PLoS Genet. 2021 Aug 30;17(8):e1009786. doi: 10.1371/journal.pgen.1009786 (PMC8432799; doi:10.1371/journal.pgen.1009786)
Supplement: S2 Table — (DOCX) [file pgen.1009786.s006.docx]

**S2 Table. Primer sequence for vector construction.**

| Vector | Primer sequence |
| --- | --- |
| Prl8a2-Luc | 5’-AGT**GCTAGC**GAATCTAGGACATCTTAAAGGAA-3’  5’-CAT**CTCGAG**TGAGTAATCGCCTCCCAACA-3’ |
| Prl8a2-CDS | 5’-ATT**GAATTC**ATGCTGCCATTGAGTCAACC-3’  5’-AT**ACTAGT**TTAGCAATCTTGCCCAGTTATGC-3’ |

Sequence labeled with bold represents the restriction site.
